# Optical density based quantification of total haemoglobin concentrations with spectroscopic optical coherence tomography

**DOI:** 10.1038/s41598-021-88063-4

**Published:** 2021-04-21

**Authors:** Carlos Cuartas-Vélez, Colin Veenstra, Saskia Kruitwagen, Wilma Petersen, Nienke Bosschaart

**Affiliations:** Biomedical Photonic Imaging Group, Faculty of Science and Technology, University of Twente, P.O. Box 217, 7500AE Enschede, The Netherlands

**Keywords:** Translational research, Near-infrared spectroscopy

## Abstract

Spectroscopic optical coherence tomography (sOCT) has emerged as a new possibility for non-invasive quantification of total haemoglobin concentrations [tHb]. Recently, we demonstrated that [tHb] measured in ex-vivo human whole-blood with a conventional sOCT system achieves a precision of 9.10 g/dL with a bias of 1.50 g/dL. This precision improved by acquiring data with a combination of focus tracking and zero-delay acquisition (FZA) that compensated for experimental limitations, increasing to 3.80 g/dL with a bias of 1.50 g/dL. Nevertheless, sOCT precision should improve at least to $\sim 2$ g/dL to be clinically relevant. Therefore, sOCT-based [tHb] determinations require the development of new analysis methods that reduce the variability of [tHb] estimations. In this work, we aim to increase sOCT precision by retrieving the [tHb] content from a numerical optimisation of the optical density (OD), while considering the blood absorption flattening effect. The OD-based approach simplifies previous two-step Lambert–Beer fitting approaches to a single step, thereby reducing errors during the fitting procedure. We validated our model with ex-vivo [tHb] measurements on flowing whole-blood samples in the clinical range (7–23 g/dL). Our results show that, with the new model, conventional sOCT can determine [tHb] with a precision of 3.09 g/dL and a bias of 0.86 g/dL compared to a commercial blood analyser. We present further precision improvement by combining the OD methodology with FZA, leading to a precision of 2.08 g/dL with a bias of 0.46 g/dL.

## Introduction

Quantification of total haemoglobin concentrations [tHb] is a key step in the diagnosis of haematologic disorders, such as anaemia^1^, haemolysis^2^ and polycythaemia^3^. Haemoglobin is a protein found inside red blood cells (RBCs) and is responsible for oxygen transportation in blood^4^. The protein binds oxygen molecules in the lungs and delivers them to cells while flowing through the body. In general, haemoglobin is classified according to its oxygen content: oxy-haemoglobin (HbO$_2$) when saturated with oxygen and deoxy-haemoglobin (Hb) when unsaturated. The total content of HbO$_2$ and Hb in blood corresponds to the [tHb]. Invasive techniques that require extraction of samples from the patient are the current standard measurements for [tHb] due to their high precision ($<0.3$ g/dL)^5^. However, for vulnerable groups, such as patients in intensive care units and premature infants where continuous monitoring is preferred, the blood collection process potentially represents a high-risk factor. Therefore, new approaches for non-invasive estimation of [tHb] have been developed.

Non-invasive [tHb] quantification methods leverage on the high absorption of haemoglobin for wavelengths in the visible and near-infrared range^6–8^. These techniques include smart-phone image analysis^9^, photoplethysmography^10^, diffuse reflectance spectroscopy^11,12^, photoacoustic spectroscopy^13,14^, ultrasound^15,16^, and more recently, spectroscopic optical coherence tomography^17,18^. Smart-phone images have grown in attention because of their accessibility, ease of use and high precision ($<3$ g/dL). However, this technology requires individual calibration to minimize subject-to-subject variability and achieve clinical precision^9^. In photoplethysmography, a photodetector measures the changes in the intensity after light interacts with tissue. In this case, the optical path followed by light is unknown, for that reason the method requires assumption-based corrections for the background absorption by any tissue structures surrounding the blood vessels of interest^10^. Photoacoustics relies on the pressure wave generated in the tissue when a pulsed-laser radiates it. If Hb and HbO$_2$ represent the main absorbers in blood, the attenuation of the wave possesses information about [tHb]. However, the concentration varies due to tissue deformation induced by pressure, thus reducing photoacoustics precision when compared to clinical standards^13^. In ultrasound, a transducer produces and receives acoustic waves that are attenuated by blood. The attenuation at different depths allows quantification of [tHb]. Even though this method has shown promising results, it is prone to errors induced by the placement of the probe, and further validation of [tHb] is still required^15^. More recently, spectroscopic optical cohere tomography (sOCT) emerged as a new possibility towards non-invasive [tHb] quantification^17,18^.

sOCT measures spectrally and depth-resolved optical properties by analyzing the spectral content of conventional optical coherence tomography (OCT) signals^19–21^. In sOCT, the spectrum of the OCT signal is split into sub-bands (windows) with a narrow localized spectral width. Each spectral window possesses depth-resolved, quantitative spectral properties of the sample. Therefore, sOCT has been used to assess absorption contrast^22,23^, quantify scattering and absorption spectra^24–26^, and evaluate local *in-vivo* chromophore concentrations^18,27,28^. More specifically, sOCT systems employing visible-light sources have shown great potential for mapping oxygen saturation (stO$_2$) based on the absorption coefficient spectrum^18,29–36^. Visible-light sOCT oximetry exploits the differences in absorption between HbO$_2$ and Hb for wavelengths within 500 and 600 nm, where the shape of the attenuation coefficient spectrum contains enough information to retrieve stO$_2$, stO$_2$ = HbO$_2$ / (HbO$_2$ + Hb). In contrast, quantification of [tHb] requires the amplitude of the spectrum in addition to its shape ([tHb] = HbO$_2$ + Hb)^17^. Therefore, [tHb] quantification is challenging, because the amplitude not only depends on the [tHb] concentration, but also on the system roll-off, the axial point spread function and the scattering by RBCs.

The feasibility of sOCT based [tHb] quantification has been demonstrated for rodents in-vivo^18^. More recently, we demonstrated [tHb] measurements for ex-vivo human whole-blood in the clinical range^17^ (7–23 g/dL). In that work^17^, we compared the performance of sOCT-based [tHb] under two different acquisition schemes: (i) conventional sOCT and (*ii*) an extension with a combination of focus tracking and zero-delay acquisition. For conventional sOCT, we accounted for attenuation related to scattering by RBCs and compensated the system-dependent attenuation. After comparison to expected values from a commercial blood analyser, conventional sOCT retrieved [tHb] with low precision (9.1 g/dL with a bias of 1.5 g/dL). To enhance the performance of sOCT, we employed an alternative experimental approach that overcame some of the physical limitations of the system. In this scheme, data acquired with a combination of focus tracking and zero-delay acquisition (FZA) improved the [tHb] precision to 3.80 g/dL with a bias of 1.50 g/dL. Simultaneously, FZA allowed the acquisition of data for higher haemoglobin concentrations ($> 18$ g/dL) compared to conventional sOCT. Despite those results, the precision of sOCT remains low in contrast to clinical standards and other clinically-relevant non-invasive techniques whose precision is $\sim 2$ g/dL^7^. Therefore, there is a need to improve on our current sOCT methodology and reduce the variability of sOCT based [tHb] estimations.

In this work, we focused on improving the precision of [tHb] estimations for both conventional sOCT and its extension with FZA. We developed a procedure to quantify [tHb] by numerical optimization of the optical density (OD) considering the absorption flattening effect in whole-blood. Our method leverages on the depth, and wavelength dependence of the OD^18,35^, enabling [tHb] estimations in a single step, in contrast to traditional Lambert-Beer fitting where the attenuation spectrum is fit from the signal depth decay and [tHb] derived from the retrieved attenuation coefficient. The OD allows simultaneous evaluation of a depth interval for the entire wavelength range, thereby reducing numerical errors induced during the two-step fitting and the variability of estimated concentrations. To illustrate the performance of our algorithm, we processed ex-vivo human blood data in the clinical range and retrieved [tHb] with conventional sOCT and FZA.

## Methods

### Visible-light spectroscopic optical coherence tomography system

**Figure 1:**
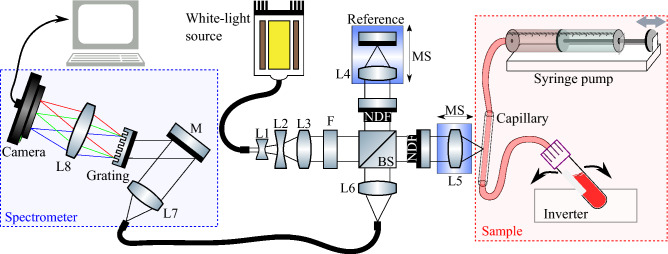
Schematic of the sOCT system to measure *ex-vivo* [tHb] in flowing whole human-blood. Light propagated through the optical system and focused into the capillary tube. A syringe pump established blood flow from a falcon tube placed on an inverter. Light backscattered by blood and reflected at the reference mirror was collected and guided to the spectrometer. *L1–L8* lenses; *F* short-pass filter; *BS* beamsplitter; *NDF* neutral density filter; *MS* motorised stage; *M* mirror.

We used a custom-built sOCT system optimized for visible light, introduced in our previous work^17,37^ and schematized in Fig. 1. Briefly, from a supercontinuum broadband source (SuperK EXTREME EXB-6, NKT Photonics, Denmark) light propagated through two neutral density filters (ND05A, Thorlabs, USA) that attenuated incoming light. Three lenses (L1: LD2746-A, L2: LD2060-A, L3: LB1471-A, Thorlabs, USA) expanded and collimated the beam before a short-pass filter (FESH0700, Thorlabs, USA) filtered out wavelengths above 700 nm. A 10:90 beam splitter (BS028, Thorlabs, USA) guided 90% of the incoming light towards the reference arm and 10% towards the sample arm. In the reference arm, a variable neutral density filter (NDC-50C-4M, Thorlabs, USA) controlled the intensity of light focused by a lens (L4: AC127-025-A, Thorlabs, USA) on the reference mirror (PF10-03-P01, Thorlabs, USA). For our FZA method, a motorized stage (T-LS13M, Zaber, USA) simultaneously displaced the reference lens and reference mirror. In the sample arm, a variable neutral density filter (NDC-50C-2M-A, Thorlabs, USA) adjusted the intensity of light focused by a lens (L5: AC127-025-A, Thorlabs, USA) into a glass capillary (Fisher Scientific B.V., The Netherlands) with an inner diameter of 1.2 mm and an outer diameter of 1.8 mm. For our FZA method, a motorized stage identical to the one in the reference arm controlled the position of the sample lens. Light reflected at the reference mirror and backscattered by blood in the sample arm combined at the beam splitter. An optic fibre guided the light into a custom-built spectrometer (HoloSpec f/1.8i, Kaiser Optical Systems, USA), where it was dispersed by a grating on a two-line scan camera (Sprint spL4096-140km, Basler, Germany) with 4096 pixels and 140 kHz line rate. The spectrometer had a spectral resolution $\delta \lambda = 0.1$ nm in the range 460–650 nm and the source spectrum had a full width at half-maximum of 100 nm centred at 550 nm, leading to a theoretical axial resolution $\ell _c\sim 1.3$ $\upmu$m in air.

### Spectroscopic optical coherence tomography signal model

#### Light–tissue interaction

Light interacting with blood experiences absorption and scattering that reduce its total intensity as a function of depth. Under the assumption of single-scattering, the Lambert–Beer law^37^ describes the intensity $I(z, \lambda )$ as a function of depth *z* and wavelength $\lambda$,

1 $$\begin{aligned} I\left( z, \lambda \right) = I_0 \left( \lambda \right) \exp \left( -2 \left( z - z_0 \right) \mu _t\left( \lambda \right) \right) , \end{aligned}$$

where $z_0$ is the initial depth position, $I_0\left( \lambda \right)$ is the intensity at the beginning of the depth interval $\left( z=z_0\right)$, and $\mu _t\left( \lambda \right)$ is the attenuation coefficient. Under this model, the intensity exponentially decreases as light propagates through the tissue. The amount of attenuation within a given depth interval is known as the optical density $\left( \text {OD}(z, \lambda )\right)$^18,33^,

2 $$\begin{aligned} \text {OD}(z, \lambda ) = \ln {\left( \frac{I\left( z,\lambda \right) }{I_0\left( \lambda \right) }\right) =\ -2\left( z-z_0\right) } \mu _t \left( \lambda \right) . \end{aligned}$$

As a measurement of attenuation, the OD is minimum at the beginning of the depth interval since light has not experienced attenuation. The OD increases proportionally with depth and attenuation coefficient as a result of more scattering and absorption events that lower the total intensity.

#### Attenuation coefficient

The attenuation coefficient $\mu _t(\lambda )$ quantifies the rate at which light is attenuated per unit length when it interacts with a medium. Under the assumption of single scattering, the total attenuation $\mu _t(\lambda ) =\mu _a(\lambda )+\mu _s(\lambda )$ arises from absorption and scattering events, which are expressed in terms of coefficients $\mu _a(\lambda )$ and $\mu _s(\lambda )$ respectively^17,37^. A power-law describes the scattering coefficient $\mu _s(\lambda )$^27,29^, $\mu _s(\lambda )=a\lambda ^{-b}$, where *b* is known as the scattering power and *a* is a scaling factor. The absorption coefficient is given by the sum of the individual absorption by each chromophore, $\mu _a (\lambda ) =\sum _{i}{C_i\mu _{a,i} (\lambda )}$, where $C_i$ and $\mu _{a,i}(\lambda )$ are the concentration and absorption spectrum of the *i*-th chromophore, respectively^27^. In the case of whole-blood, absorption is mainly due to HbO$_2$ and Hb, and their absorption coefficient spectra ($\mu _{a, HbO_2}$ and $\mu _{a, Hb}$ respectively) have been documented in the literature^8^. Under these assumptions for scattering and absorption, the attenuation coefficient corresponds to

3 $$\begin{aligned} \mu _t\left( \lambda \right) =a\lambda ^{-b}+\sum _{i} C_i\mu _{a,i}\left( \lambda \right) . \end{aligned}$$

For the absorption coefficient, we considered the effective absorption of inhomogeneously distributed chromophores. In the case of blood, photons bypassing RBCs experience no absorption by haemoglobin. This absorption inhomogeneity causes a non-linear scaling between absorption and concentration, therefore, the *effective absorption coefficient* ${\hat{\mu }}_{a,i}(\lambda )$ for whole-blood is corrected as^8^

4 $$\begin{aligned} {\hat{\mu }}_{a,i}(\lambda ) = \frac{1 - \exp \left( -C_i\mu _{a,i}(\lambda ) d_{RBC}\right) }{d_{RBC}}, \end{aligned}$$

where $d_{RBC}$ is the width of an RBC under a cube approximation $d_{RBC} \sim 4.48$ $\upmu$m^8^.

### Blood samples and data processing

#### Human blood samples

We processed an existing set of data that was acquired from ex-vivo human whole-blood samples, initially analysed in our previous work^17^. Measurement setup and acquisition settings were thus identical to our previous work. Briefly, blood samples were collected and prepared from six healthy donors from the Experimental Centre for Technical Medicine of the University of Twente. The local Medical Research Ethics Committee (METC Twente now part of MEC-U) approved the blood collection procedure, and all procedures followed relevant guidelines and regulations. All volunteers gave written informed consent for data usage following the guidance of the Declaration of Helsinki. From donated blood, we prepared a set of samples with different [tHb]. We mixed donated blood with PBS to reduce [tHb] or removed blood plasma to increase it and added heparin to prevent coagulation. Falcon tubes filled with 9 mL of blood were placed on an inverter with a frequency of 0.1 Hz to avoid sedimentation. A syringe pump (AL1000-220, World Precision Instruments, USA) established a blood flow of 0.3 mL/min in the glass capillary. The capillary had a small angle $\theta \sim 10^\circ$ relative to the incident beam (Fig. 1).

We created a set of 23 blood samples with a varying concentration in the range 7.0–23.0 g/dL. The 23 samples were measured with FZA and 21 of those samples measured with conventional sOCT. We obtained validation values for expected [tHb] from three measurements on each sample with a blood analyser (Avoximeter 1000E, Instrumentation Laboratory, USA) and calculated the mean and standard deviation (SD) in each case. By using sOCT, we also estimated [tHb] for three measurements per sample with respective mean and SD. We excluded some samples and measurements from our study. For conventional sOCT, two samples with high concentrations (expected [tHb] 19.1 g/dL and 21.5 g/dL) were excluded because of the low recorded signal, which was likely due to high absorption by blood. Another sample for conventional sOCT was excluded (expected [tHb] 12.6 g/dL) after finding a reflection artefact caused by the capillary. A total of 18 from the 21 sample data were used in our conventional sOCT analysis. From the triplo measurements on 18 samples, we omitted two measurements for an expected [tHb] $=9.1$ g/dL and one measurement for an expected [tHb] $=19.4$ g/dL since no scattering was observed, presumably because of low blood flow in the capillary. For FZA, one measurement for an expected [tHb] $= 19.4$ g/dL was omitted after finding no scattering as in the conventional case. The samples with excluded measurements are indicated and specified in the results section.

#### Conventional sOCT

**Figure 2:**
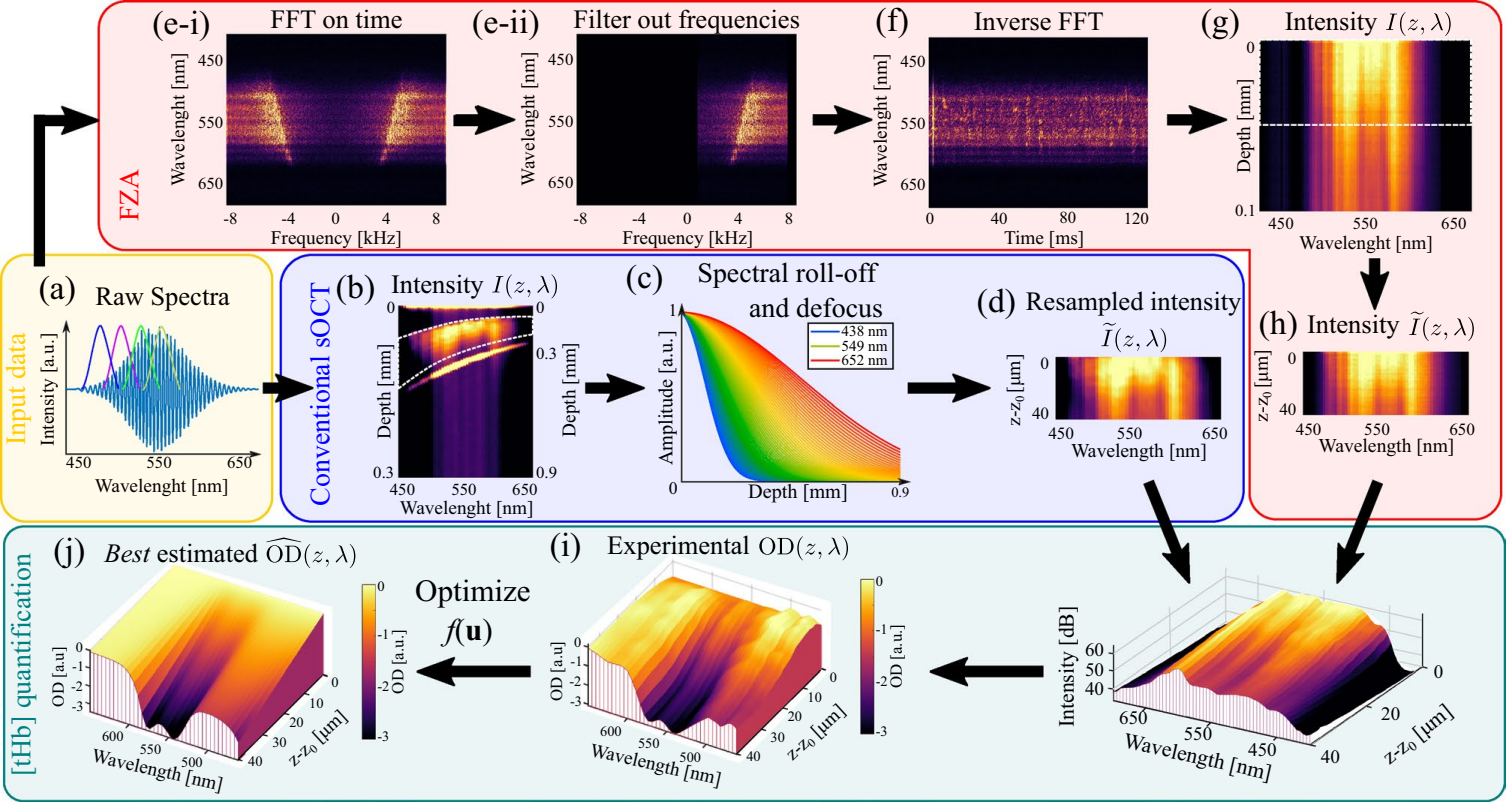
Estimation of [tHb] from sOCT data. **(a)** Spectra acquired with the sOCT system were processed according to the acquisition scheme: conventional sOCT (blue path **(b–d)**) and FZA (red path **(e–g)**). For conventional sOCT, spectral analysis **(b)** retrieved the intensity as a function of depth and wavelength $I(z, \lambda )$. We compensated the system roll-off and the defocus, and resampled the intensity **(d)** within the region of interest ${\widetilde{I}}(z, \lambda )$. For FZA, spectra were Fourier transformed into the frequency domain **(e-i)**, negative frequencies were filtered out **(e-ii)**, and data was transformed back to the time domain **(f)**. The spectral analysis yielded the intensity as a function of wavelength and depth **(g)**. Since the translation stage controlled the optical path length resampling was not required **(h)**. In both cases, we selected a region of interested of 40 $\upmu$m in depth, the experimental OD **(i)** was calculated with Eq. (2) and numerically estimated $\widehat{\text {OD}}$ **(j)** with Eqs. (5) and  (6).

From the ex-vivo blood samples, we acquired a set of 2500 spectra consisting of 4096 data points. Each spectrum had an exposure time of 50 $\upmu$s and was measured at a line rate of 16.7 kHz. We retrieved the background spectrum at a depth of 1 mm inside the blood sample, where blood had attenuated all incoming light and subtracted the background from the raw spectra. Then, we performed a spectral analysis for each measurement as depicted in Fig. 2. Short-time Fourier transforms (STFTs) were taken with a Gaussian sub-band window of 20 nm ($e^{-2}$ width) with 75% overlap over the entire spectral range (Fig. 2a, blue path). The resulting signal represented the intensity as a function of depth and (sub-band) wavelength $I(z,\lambda )$ (Fig. 2b). In each STFT, the maximum imaging depth $z_{max}$ depends on the window central-wavelength $\lambda _0$, $z_{max} = \lambda _0^2 / 4n\delta \lambda$, where *n* is the index of refraction (assumed as $n = 1.4$) and $\delta \lambda$ is the spectral resolution. Consequently, the maximum imaging depth was $\sim 300$ $\upmu$m at 450 nm and $\sim 900$ $\upmu$m at 650 nm.

Subsequently, we compensated for any signal losses due to system attenuation, which included a correction for the system roll-off and the axial point spread function of the sample lens (Fig. 2c). The system roll-off is a decrease in the OCT signal as a function of depth caused by the finite resolution of the spectrometer. Compensation for the system roll-off has recently shown improvements in the fitting of stO$_2$ based on sOCT^38^. Therefore, we applied a correction as described by Rubinoff et al.^38^. We also compensated for the signal losses by the axial point spread function (hereafter referred to as defocus) of the sample lens with the model described by Almasian et al.^39^. Next, we estimated the experimental OD in a region of interest (ROI) defined in the depth range 80–120 $\upmu$m, at a location $z_0 = 15$ $\upmu$m inside the blood sample. To use the same ROI for all wavelengths, we varied the number of data points within the ROI from 368 at 450 nm to 82 at 650 nm (white dashed lines in Fig. 2b). We equalized the number of data points in the ROI by over-sampling the intensity values to 2048 points across the spectral range, $I(z, \lambda ) \rightarrow {\widetilde{I}}(z,\lambda )$ (Fig. 2d). By using ${\widetilde{I}}(z,\lambda )$ and Eq. (2), the experimental OD for each spectrum was retrieved (Fig. 2i, green path).

In order to quantify [tHb], we computed a numerical estimate $\widehat{\text {OD}}(z,\lambda )$ of the experimental OD by combining Eqs. (2)–(4) into

5 $$\begin{aligned} \widehat{\text {OD}}(z, \lambda ) = -2\left( z-z_0\right) \left( a\lambda ^{-b} + \frac{2 - \exp \left( -C_{Hb} \mu _{a,Hb} (\lambda ) d_{RBC}\right) - \exp \left( -C_{HbO_2}\mu _{a,HbO_2}(\lambda ) d_{RBC} \right) }{d_{RBC}} \right) + \alpha , \end{aligned}$$

where $C_{HbO_2}$ and $C_{Hb}$ are the concentrations of HbO$_2$ and Hb respectively, and $\alpha$ is a background term. The unknown set of parameters ${\mathbf {u}}=\left( C_{HbO_2}, C_{Hb}, a, b, \alpha \right)$ was determined with a non-linear gradient-based optimisation method, i.e. by minimizing an error function $f({\mathbf {u}})$ defined as the squared difference between the experimental and estimated ODs,

6 $$\begin{aligned} \min _{{\mathbf {u}}} \ f({\mathbf {u}}) = \sum _z \sum _\lambda \left| \ln {\left( \frac{{\widetilde{I}}\left( z,\lambda \right) }{{\widetilde{I}}_0\left( \lambda \right) }\right) } - \widehat{\text {OD}}(z, \lambda )\right| ^ 2. \end{aligned}$$

We set a seed of zeros for all parameters ${\mathbf {u}} = 0$ with boundaries between zero and infinity $0\le {\mathbf {u}} < \infty$. Since this is a generic optimization problem, we optimized the error function with the Matlab solver *fmincon*, and used as stop criteria a function tolerance of $10^{-10}$, a tolerance on the current point of $10^{-10}$ and a limit of $2\times 10^4$ iterations. Once convergence had been reached, the retrieved OD was the best estimate of the experimental OD, $\widehat{\text {OD}}(z, \lambda ) \simeq \text {OD}(z, \lambda )$ (Fig. 2j) and the [tHb] corresponded to the sum of the outputs $C_{Hb}+C_{HbO_2}$.

#### Focus tracking and zero-delay acquisition

We also processed data acquired with FZA, introduced in our previous work^17,37,40^. In this acquisition scheme, the signal losses due to system attenuation (roll-off and defocus) are compensated experimentally during acquisition. Optimal sensitivity in depth and independence on system attenuation is achieved by translation and oscillation of the reference mirror, while simultaneously, displacing the imaging lens in the sample arm. The periodic movement of the reference mirror induced a Doppler shift of the sOCT signal with frequency $f_D = 2 v_R/\lambda _0$, where $v_R = 0.85$ mm/s is the velocity of the reference mirror and $\lambda _0 = 550$ nm is the central wavelength of the spectrum. The motorized stages (Fig. 1) displaced the sample lens and reference mirror in steps of 2 $\upmu$m across 100 $\upmu$m inside the blood sample. At each step, we acquired 2500 spectra with 4096 data points, with an exposure time of 50 $\upmu$s and at a line rate of 16.7 kHz. At a depth of 1 mm, the background was measured and subtracted from each spectrum.

The acquired spectra (Fig. 2a, red path) were Fourier transformed from the temporal into frequency domain (Fig. 2e–i). A band-pass filter filtered out the complex conjugate in the negatively shifted frequencies while maintaining those between 0.4 and 7.3 kHz. The filtered signal was transformed back into the temporal domain (Fig. 2f). We performed the spectral analysis at each step with a Gaussian spectral window of 5 nm ($e^{-2}$ width) without overlapping (Fig. 2g). We decreased the window width compared to conventional sOCT in order to obtain the most stable outcome for both methods^37^. We retrieved the experimental OD within a depth interval of 40 $\upmu$m (white dashed box in Fig. 2g). Since this depth interval was controlled by the motorized stage in the reference arm, no resampling was required ${\widetilde{I}}(z, \lambda ) = I(z, \lambda )$ (Fig. 2h). We derived the [tHb] by numerical optimization of Eq. (6) with the same parameters as for conventional sOCT (Fig. 2i,j).

### Comparison to previous work

We compared our OD-based method for conventional sOCT and FZA to our previous work, in which we processed exactly the same set of data with a Lambert-Beer based model^17^. In that work, we estimated the [tHb] in a two-step procedure, where the attenuation coefficient spectra were retrieved with a Lambert-Beer fit on the depth- and wavelength dependent intensity data in the first step, and the [tHb] concentration was retrieved from a fit on the attenuation coefficient in the second step. Details on this analysis procedure can be found in Ref.^17^. For the sake of comparison, our methods above relied on identical analysis settings as in our previous work: initial depth position, fitting depth range, total acquisitions, bandwidth, frequency windows, spectral window width and number of spectral windows.

## Results

### Optical density estimation

#### Conventional sOCT

**Figure 3:**
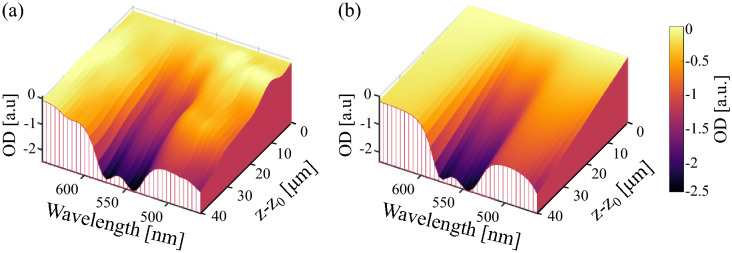
Comparison between the ODs obtained with conventional sOCT for an expected [tHb] $= 16.9$ g/dL with a coefficient of determination $R^2 = 0.97$. **(a)** Experimental OD and **(b)** estimated $\widehat{\text {OD}}$ after the algorithm reached convergence.

We estimated the OD of 18 human whole-blood samples and a total of 51 measurements with conventional sOCT. Figure 3 presents a comparison between the experimental OD (a) and estimated $\widehat{\text {OD}}$ (b) after the algorithm had reached convergence. At the beginning of the depth interval, $z = z_0$, the OD was minimum and varied as a function of depth and wavelength. Speckle caused fluctuations in the experimental OD. From the retrieved $\widehat{\text {OD}}$ in Fig. 3b, we obtained a [tHb] of 16.2 g/dL with a coefficient of determination $R^2 = 0.97$ for a sample with an expected [tHb] of 16.9 g/dL. After the 51 measurements were processed, we obtained an average coefficient of determination with ± SD, $R^2 = 0.79 \pm 0.25$.

#### Focus tracking and zero-delay acquisition

**Figure 4:**
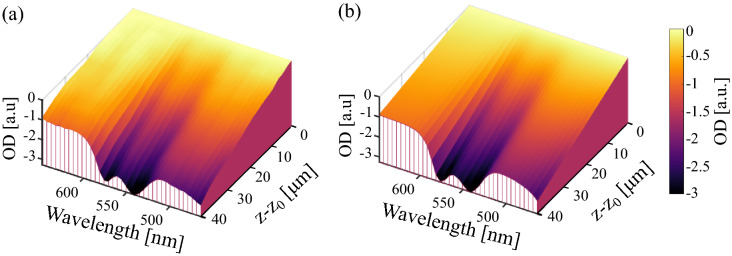
Estimation of the ODs achieved by FZA for an expected [tHb] $= 16.9$ g/dL with a coefficient of determination $R^2 = 0.98$. **(a)** Experimental OD and **(b)** estimated $\widehat{\text {OD}}$ after the algorithm reached convergence.

We repeated the same procedure for the 23 human whole-blood samples and a total of 68 measurements acquired with FZA. Figure 4 shows a comparison of the experimental OD (a) and estimated $\widehat{\text {OD}}$ (b), for the same sample as for the conventional sOCT result in Fig. 3 (expected concentration 16.9 g/dL). In this case, we retrieved a [tHb] of 16.5 g/dL with an $R^2 = 0.98$. After processing the 68 measurements we obtained an average coefficient of determination with ± SD, $R^2 = 0.97 \pm 0.04$.

### Total haemoglobin concentration

**Figure 5:**
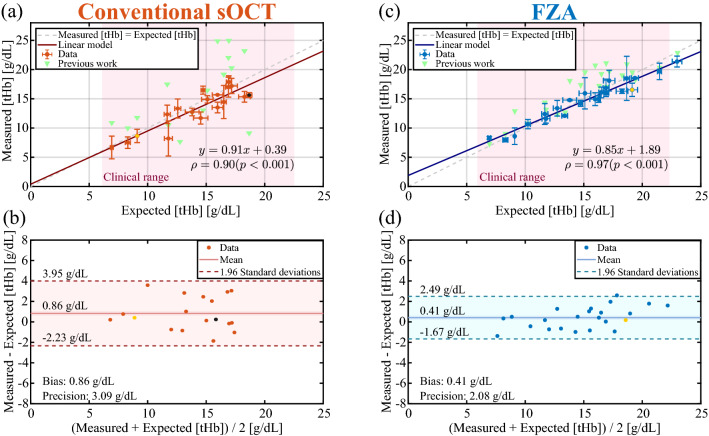
Total haemoglobin concentrations retrieved after processing all data sets. **(a)** Measured [tHb] as a function of expected [tHb] with conventional sOCT (mean value ± SD) for all measurements and **(b)** Bland–Altman plot for the same data from which we obtain a bias of 0.86 g/dL and a precision of 3.09 g/dL. **(c)** Measured [tHb] as a function of expected [tHb] with FZA for all measurements and **(d)** Bland–Altman plot for the same data, showing a bias of 0.41 g/dL with a precision of 2.08 g/dL. The points filled with yellow had one excluded measurements, while those filled in black had two exclusions. For comparison purposes, data form our previous work^17^ is included in **(a,c)**. For visualization clarity, the SDs of previous data are omitted from **(a,c)**, and previous data points not included in **(b,d)**.

The measured [tHb] as a function of expected [tHb] is presented in Fig. 5. We have added the mean retrieved [tHb] from our previous work^17^ for comparison purposes (omitting SDs for visualization clarity). Conventional sOCT is shown in Fig. 5a for which a linear regression yields $y = 0.91x + 0.39$ with Pearson correlation coefficient $\rho = 0.90$ and $p < 0.001$. From the Bland–Altman plot (Fig. 5b), we obtained a bias of 0.86 g/dL and a precision of 3.09 g/dL (defined as 1.96 SDs). For FZA, the measured [tHb] as a function of expected [tHb] is presented in Fig. 5c, leading to a linear regression $y = 0.85x + 1.89$ and Pearson correlation coefficient $\rho = 0.97$ with $p < 0.001$. Figure 5d is the Bland–Altman plot that yields a bias of 0.41 g/dL and a precision of 2.08 g/dL (1.96 SDs). For all plots, samples with one measurement excluded are filled in yellow and those with two measurements excluded are filled in black.

## Discussion

In this work, we aimed to improve the precision of retrieved [tHb] based on sOCT. We quantified [tHb] from ex-vivo human whole-blood based on a numerical estimation of the OD for two acquisition schemes, conventional sOCT and an extension with FZA. We related the [tHb] directly to the OD and considered the blood flattening effect in the signal model. These factors together increase the robustness of [tHb] estimations and reduce the variability across sOCT measurements, thereby improving the precision and bias of sOCT-based [tHb] determinations compared to our previous work^17^.

With our new model, we processed a set of samples acquired with conventional sOCT and FZA. Table 1 presents a comparison of the results in terms of precision and bias for conventional sOCT and FZA from this work (OD-based) and our prior work on exactly the same set of data (Lambert–Beer fit). With the new model, we obtained a precision of 3.09 g/dL, 2.9 times higher than our previous work, where we achieved 9.10 g/dL. We retrieved a bias of 0.86 g/dL, representing a decrease with a factor of 1.8 from 1.50 g/dL in our prior work. From the 23 samples measured with FZA, we achieved a precision of 2.08 g/dL, 1.8 times higher than the 3.80 g/dL previously obtained. Similarly, the bias was 0.41 g/dL being 3.7 times lower than 1.50 g/dL in our former work.

**Table 1:** Comparison of the precision and bias obtained for multiple sOCT-based [tHb] estimations.

Parameter	Acquisition	Lambert–Beer fit	OD-based
Bias (g/dL)	Conventional sOCT	1.50	0.86
	FZA	1.50	0.41
Precision (g/dL)	Conventional sOCT	9.10	3.09
	FZA	3.80	2.08

The main advantage of our technique is the simplification in the data processing to retrieve [tHb]. Specifically, the traditional Lambert-Beer fit requires a two-step procedure, where the attenuation coefficient spectrum is fit from the intensity data and the [tHb] estimated from the retrieved attenuation. In contrast, our OD-based method determines [tHb] in a single-step by relating the signal attenuation directly to the OD, leveraging on its depth and wavelength dependence. This procedure reduces the errors ascribed to the two-step fitting. Our method builds upon the methodology employed for mapping stO$_2$ in rodents^18,35^. The stO$_2$ estimation directly relates the Hb and HbO$_2$ content in blood to the OD. We considered the blood flattening effect in the signal model and applied that methodology in human whole-blood samples under two different acquisition schemes. In addition, this paper demonstrates a direct comparison between the outcomes of the Lambert–Beer model and the OD-based model, which aids to a more in depth understanding of the added value of the OD model.

**Figure 6:**
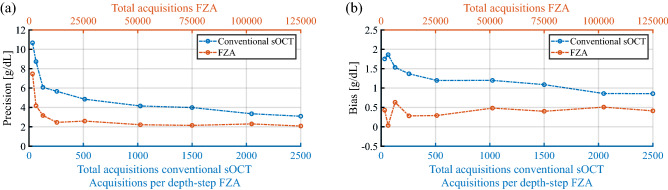
Precision **(a)** and bias **(b)** a function of acquisitions for conventional sOCT and FZA. For conventional sOCT, the precision and bias improve with increasing acquisitions. For FZA the precision and bias converge after 250 acquisitions per depth step (a total of 12, 500 acquisitions).

Conventional sOCT and its extension with FZA have a high linear correlation between measured and expected [tHb] given by the Pearson correlation coefficient of 0.90 and 0.97, respectively. From our results, we conclude that FZA is more precise than conventional sOCT in terms of [tHb] bias and precision, similarly to our previous work where we showed improved performance with FZA. With an average coefficient of determination $R^2 = 0.97 \pm 0.04$, FZA has a lower deviation between estimated and experimental data compared to $R^2 = 0.79 \pm 0.25$ obtained for conventional sOCT. This is further strengthened by visual inspection of Figs. 3 and  4 generated from a sample with the same concentration and similar $R^2$. We hypothesise that the intensity fluctuations along the depth axis in the conventional method (Fig. 3) are attributable to speckle. That may also explain why the relative improvement on the precision of the [tHb] determination is higher for conventional sOCT compared to FZA. Since the OD considers the entire depth and wavelength range, intensity fluctuations attributable to speckle should be less severe. However, further tests to analyse the impact of speckle in [tHb] estimation is still required. As presented in Fig. 6, the precision and bias for conventional sOCT improve with an increasing number of acquisitions. Since the number of acquisitions reduces the speckle contrast, it is expected that more acquisitions will further improve the outcome for conventional sOCT, similar to the reduction in speckle contrast with increased averaging^41^. Advanced speckle reduction techniques specifically developed for sOCT can potentially further enhance the results for conventional sOCT^42–44^.

FZA needs a set of temporal measurements to sample the oscillation of the reference mirror at each depth step. Therefore, the acquisition time increases with the number of depth-steps, the depth interval and the set of measurements. In this study, we acquired 2500 measurements at each depth-step (corresponding to an acquisition time of 150 ms) and scanned a depth interval of 100 $\mu$m with a total of 50 depth steps. As presented in Fig. 6, increasing the total number of acquisitions for FZA makes the precision and bias converge to the reported values after $\sim 250$ acquisitions at each depth step (a total of 12, 500 acquisitions). For this number of acquisitions, the temporal sampling is sufficient to correctly characterize the mirror oscillation, and although more acquisitions produce higher temporal sampling, it does not represent an improvement in terms of precision or bias. Our results demonstrate that the [tHb] can be derived within a depth interval of 40 $\upmu$m with 250 acquisitions (15 ms), potentially increasing the acquisition speed for FZA. The eventual choice for conventional sOCT or FZA will depend on required measurement speed, total [tHb] range and precision. Conventional sOCT may be preferred when high-speed imaging is required, at the cost of measurement precision. As shown in Fig. 5, the applicability of conventional sOCT is limited for high [tHb] concentrations. When high [tHb] concentrations are involved and optimal precision is required, FZA represents a more attractive approach.

For the implementation of the OD model, we used a gradient-based optimizer instead of a least-square fitting algorithm. Although it is possible to use both optimisers, we found that the outcome of the gradient-based method was more consistent for all the data sets, since it produced the highest average coefficient of determination. Whereas the gradient-based algorithm is prone to local minima that may reduce its repeatability, the depth interval and wavelength range can be adjusted to produce the most stable outcome. In our study, we found that a depth interval of 40 $\upmu$m and a wavelength range of 450–650 nm yielded a reliable output. Those ranges can be fine tuned further, since the main absorption features of Hb and HbO$_2$ take place within 500–600 nm, and the depth interval selection may influence [tHb] outcomes.

In our current OD-based model, we made several assumptions concerning light-blood interaction. First of all, we incorporated a single-scattering approach that limits the depth interval selection and we simplified the wavelength-dependent behaviour of RBC-scattering to a power function. Secondly, we assumed ideal laminar blood flow and homogeneous distribution of RBCs throughout the capillary. Because of the non-Newtonian nature of blood, blood flow can induce an inhomogeneous distribution of RBCs inside the measurement capillary. Therefore, the quantified [tHb] may vary at different locations. Other phenomena such as RBC aggregation due to the low shear rate at the capillary wall and sedimentation related to gravity may also influence the measurements of [tHb]. Therefore, future models that account for complex light-blood interaction phenomena can potentially further improve the performance of sOCT.

For future in-vivo application of our method, we expect that blood vessels with a diameter of $\ge 50$ $\upmu$m are most appropriate, taking the cell-free layer adjacent to the vessel wall into account^37^. For the in-vivo experiment, blood vessel depth with respect to the skin surface will affect precision due to other sources of multiple scattering, signal attenuation by surrounding tissue and—for conventional sOCT—signal roll-off and defocus. From our experiments, we expect that our method performs best at superficial blood vessels ($\le 300$ $\upmu$m deep) with a diameter $\ge 50$ $\upmu$m. These vessels can be found in in the forearm or sublingual tissue^45,46^. The optimal body location for *in-vivo* [tHb] determinations will be a priority for future research.

Our results demonstrate the potential applicability of sOCT for [tHb] determinations with clinically-relevant precision. Thereby, this work presents an important contribution to the future development of in-vivo [tHb] quantification by sOCT.

## Conclusion

In conclusion, we developed and applied a novel processing scheme to obtain [tHb] concentrations with sOCT. Our approach leverages on numerical optimisation of the OD considering the blood flattening effect. Our method directly relates the [tHb] to the OD, thus estimating the [tHb] in a single step. We validated our methodology with ex-vivo human whole-blood data under two acquisition schemes: conventional sOCT and an extension with FZA. In both cases, we obtained a high correlation between the expected [tHb] from a commercial blood analyser and our estimations with sOCT, as indicated by the Pearson correlation coefficient of 0.90 and 0.97 for conventional sOCT and FZA, respectively. For conventional sOCT, we obtained a precision of 3.09 g/dL and a bias of 0.86 g/dL, representing an improvement with a factor of 2.9 and 1.8 respectively compared to our previous work. We achieved a precision of 2.08 g/dL with a bias of 0.41 g/dL for data acquired with FZA. In this case, the results improved in a factor of 1.8 and 3.7 for the precision and the bias, respectively.

## Data Availability

The data sets and algorithms generated and analysed for this manuscript are available by contacting the corresponding author upon prior request.
